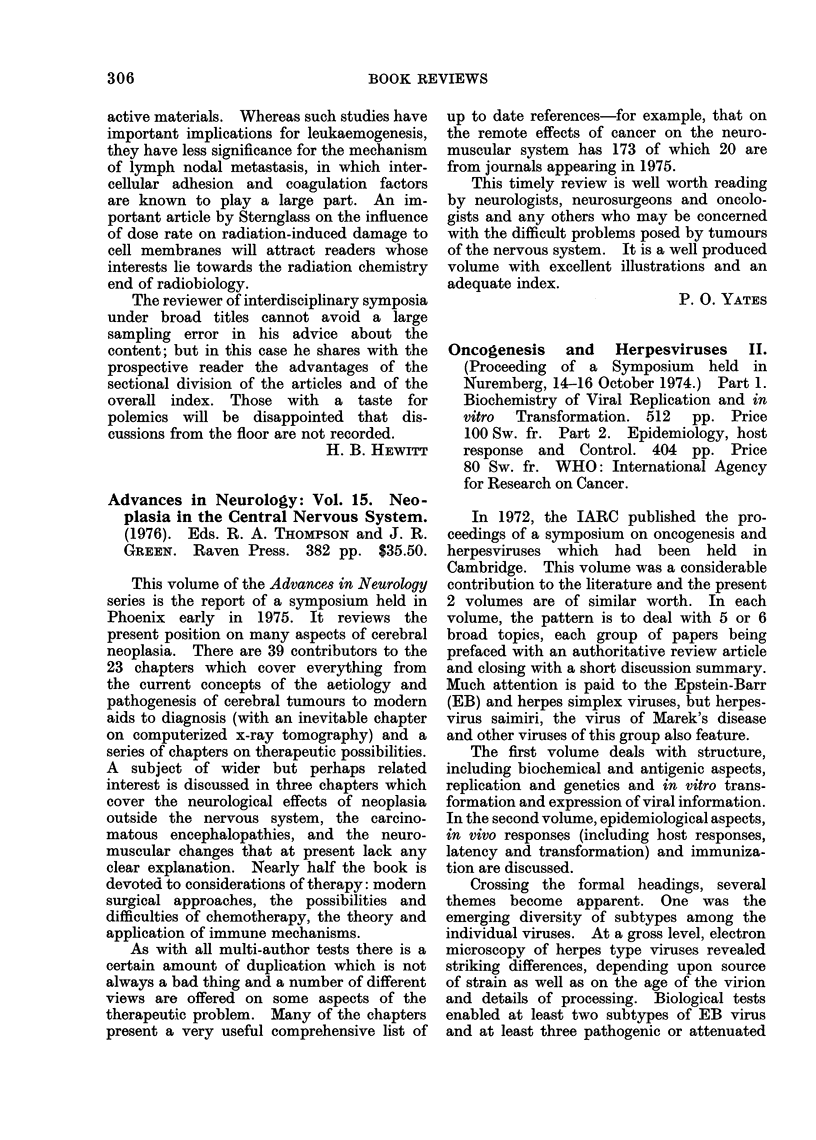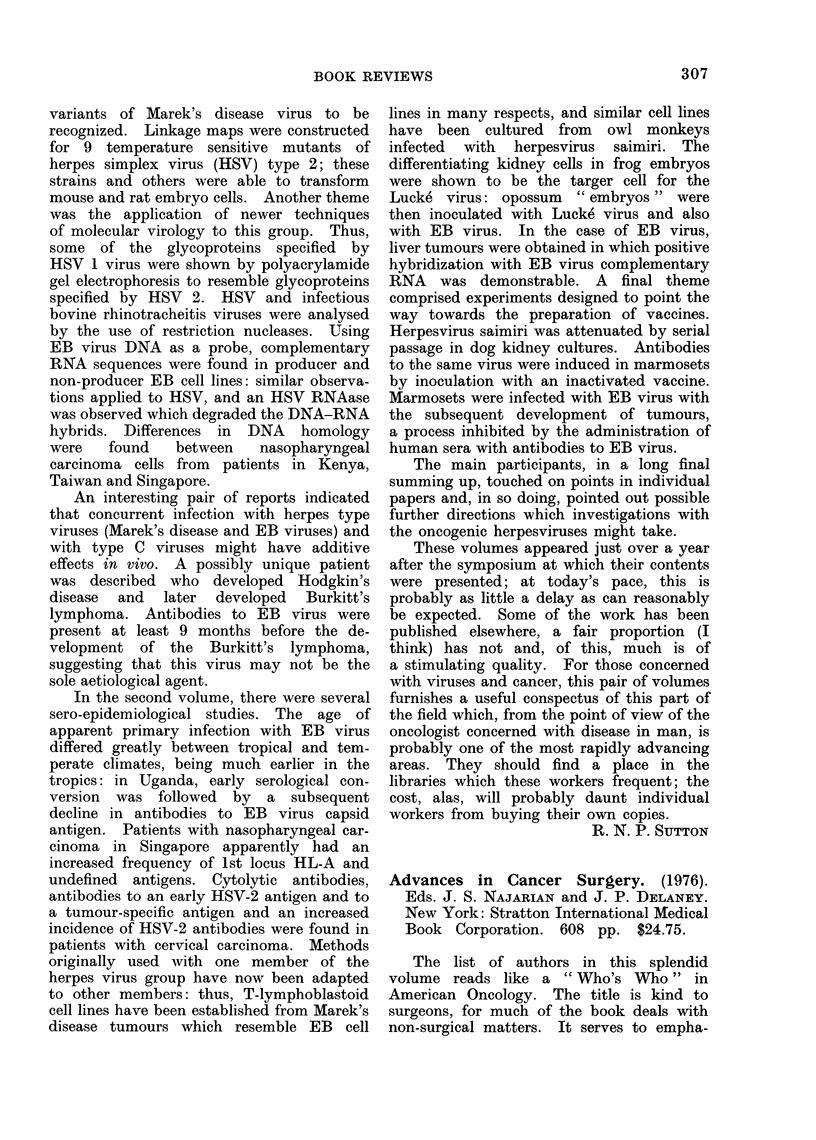# Oncogenesis and Herpesviruses II

**Published:** 1976-09

**Authors:** R. N. P. Sutton


**Oncogenesis and Herpesviruses II.** (Proceeding of a Symposium held in Nuremberg, 14–16 October 1974.) Part 1. Biochemistry of Viral Replication and *in vitro* Transformation. 512 pp. Price 100 Sw. fr. Part 2. Epidemiology, host response and Control. 404 pp. Price 80 Sw. fr. WHO: International Agency for Research on Cancer.

In 1972, the IARC published the proceedings of a symposium on oncogenesis and herpesviruses which had been held in Cambridge. This volume was a considerable contribution to the literature and the present 2 volumes are of similar worth. In each volume, the pattern is to deal with 5 or 6 broad topics, each group of papers being prefaced with an authoritative review article and closing with a short discussion summary. Much attention is paid to the Epstein-Barr (EB) and herpes simplex viruses, but herpesvirus saimiri, the virus of Marek's disease and other viruses of this group also feature.

The first volume deals with structure, including biochemical and antigenic aspects, replication and genetics and *in vitro* transformation and expression of viral information. In the second volume, epidemiological aspects, *in vivo* responses (including host responses, latency and transformation) and immunization are discussed.

Crossing the formal headings, several themes become apparent. One was the emerging diversity of subtypes among the individual viruses. At a gross level, electron microscopy of herpes type viruses revealed striking differences, depending upon source of strain as well as on the age of the virion and details of processing. Biological tests enabled at least two subtypes of EB virus and at least three pathogenic or attenuated variants of Marek's disease virus to be recognized. Linkage maps were constructed for 9 temperature sensitive mutants of herpes simplex virus (HSV) type 2; these strains and others were able to transform mouse and rat embryo cells. Another theme was the application of newer techniques of molecular virology to this group. Thus, some of the glycoproteins specified by HSV 1 virus were shown by polyacrylamide gel electrophoresis to resemble glycoproteins specified by HSV 2. HSV and infectious bovine rhinotracheitis viruses were analysed by the use of restriction nucleases. Using EB virus DNA as a probe, complementary RNA sequences were found in producer and non-producer EB cell lines: similar observations applied to HSV, and an HSV RNAase was observed which degraded the DNA–RNA hybrids. Differences in DNA homology were found between nasopharyngeal carcinoma cells from patients in Kenya, Taiwan and Singapore.

An interesting pair of reports indicated that concurrent infection with herpes type viruses (Marek's disease and EB viruses) and with type C viruses might have additive effects *in vivo*. A possibly unique patient was described who developed Hodgkin's disease and later developed Burkitt's lymphoma. Antibodies to EB virus were present at least 9 months before the development of the Burkitt's lymphoma, suggesting that this virus may not be the sole aetiological agent.

In the second volume, there were several sero-epidemiological studies. The age of apparent primary infection with EB virus differed greatly between tropical and temperate climates, being much earlier in the tropics: in Uganda, early serological conversion was followed by a subsequent decline in antibodies to EB virus capsid antigen. Patients with nasopharyngeal carcinoma in Singapore apparently had an increased frequency of 1st locus HL-A and undefined antigens. Cytolytic antibodies, antibodies to an early HSV-2 antigen and to a tumour-specific antigen and an increased incidence of HSV-2 antibodies were found in patients with cervical carcinoma. Methods originally used with one member of the herpes virus group have now been adapted to other members: thus, T-lymphoblastoid cell lines have been established from Marek's disease tumours which resemble EB cell lines in many respects, and similar cell lines have been cultured from owl monkeys infected with herpesvirus saimiri. The differentiating kidney cells in frog embryos were shown to be the targer cell for the Lucké virus: opossum " embryos " were then inoculated with Lucké virus and also with EB virus. In the case of EB virus, liver tumours were obtained in which positive hybridization with EB virus complementary RNA was demonstrable. A final theme comprised experiments designed to point the way towards the preparation of vaccines. Herpesvirus saimiri was attenuated by serial passage in dog kidney cultures. Antibodies to the same virus were induced in marmosets by inoculation with an inactivated vaccine. Marmosets were infected with EB virus with the subsequent development of tumours, a process inhibited by the administration of human sera with antibodies to EB virus.

The main participants, in a long final summing up, touched on points in individual papers and, in so doing, pointed out possible further directions which investigations with the oncogenic herpesviruses might take.

These volumes appeared just over a year after the symposium at which their contents were presented; at today's pace, this is probably as little a delay as can reasonably be expected. Some of the work has been published elsewhere, a fair proportion (I think) has not and, of this, much is of a stimulating quality. For those concerned with viruses and cancer, this pair of volumes furnishes a useful conspectus of this part of the field which, from the point of view of the oncologist concerned with disease in man, is probably one of the most rapidly advancing areas. They should find a place in the libraries which these workers frequent; the cost, alas, will probably daunt individual workers from buying their own copies.

R. N. P. SUTTON